# Change of Outpatient Oral Surgery during the COVID-19 Pandemic: Experience of an Italian Center

**DOI:** 10.1155/2020/8893423

**Published:** 2020-07-22

**Authors:** Francesco Bennardo, Alessandro Antonelli, Selene Barone, Michele Mario Figliuzzi, Leonzio Fortunato, Amerigo Giudice

**Affiliations:** School of Dentistry, Department of Health Sciences, Magna Graecia University of Catanzaro, Viale Europa, Catanzaro 88100, Italy

## Abstract

COVID-19, which appeared to originate in China in December 2019, has spread worldwide in a pandemic way. The aim of this work is to present a protocol to standardize the outpatient oral surgery activities through remote triage, diagnostic tests, protections, and precautions that allow to provide care while minimizing risk for both patients and surgeons. This article summarizes the clinical and surgical experience of the Oral Surgery Unit of the “Mater Domini” Hospital (Magna Graecia University of Catanzaro, Italy) during the COVID-19 pandemic. The application of a scrupulous triage protocol, the use of remote consultations to limit patients' access to the clinic, and the correct use of PPE prevented transmission of the virus between patients and staff members.

## 1. Introduction

A novel coronavirus (severe acute respiratory syndrome coronavirus 2, SARS-CoV-2; previously known as 2019-nCoV) emerged in Wuhan (Hubei, China) in December 2019 and spread rapidly across the planet [1–4]. Several coronaviruses can cause light respiratory disease in humans, but SARS-CoV-2 can cause pneumonia, called corona virus disease-2019 (COVID-19) that might result in death due to massive alveolar damage and progressive respiratory failure [1]. COVID-19 is clinically manifested by fever, cough, dyspnea up to respiratory failure. Clinical management is principally symptomatic treatment, but severe cases require respiratory assistance with organ support in intensive care for seriously ill patients. Most of these patients are over the age of 60 and have comorbidities. No specific antiviral treatment exists, but antiviral, antimalarial, and biological drugs against other diseases were administered in clinical trials [2].

The World Health Organization declared COVID-19 a pandemic on March 12, 2020. Spain, Italy, and the United Kingdom are the three most affected European countries [5].

The person-to-person transmission routes of SARS-CoV-2 included direct transmission, through droplets and saliva (cough, sneeze, inhalation, etc.), and contact transmission with oral, nasal, and eye mucous membranes. Even the fecal-oral route could allow the transmission of the virus [6].

Transmission within healthcare workers has been documented, and the risk of cross infection may be higher between oral healthcare providers and patients due to characteristic of procedures performed in the oral cavity [7]. These data were underlying in a document published on March 15, 2020, on New York Times, that it showed dentists, dental assistants, oral and maxillofacial surgeons, and dental hygienists are the healthcare workers with the highest infection risk (exposure percentage between 95 and 100%) [8]. SARS-CoV-2 can persist in aerosols for 3 hours and up to 72 hours on selected surfaces [9, 10].

In March 2020, based on the severity of the pandemic, the Italian Ministry of Health ordered the suspension of all nonurgent outpatient activities in hospitals and clinics until further notice, including private health service providers [11].

The aim of this work is to present a protocol to standardize the outpatient oral surgery activities through remote triage, diagnostic tests, protections, and precautions that allowed to provide care while minimizing risk for both patients and surgeons.

## 2. Materials and Methods

The authors reported the experience of the Oral Surgery Unit of the “Mater Domini” Hospital affiliated to the School of Dentistry of Magna Graecia University of Catanzaro (Catanzaro, Italy). According to the Declaration of Helsinki on medical protocol and ethics, the regional Ethical Review Board of Central Calabria approved this retrospective report. Informed consent was obtained from all patients admitted to our unit.

### 2.1. Concept of Urgent and Postponable Procedures during Pandemic

Outpatient oral surgery procedures were divided into the following categories:

(i)  Postponable procedures
  - Periodic oral examinations and recall visits
  - Extraction of asymptomatic teeth
  - Implantology
(ii) Pathologies responsive to medical therapy
  - Osteonecrosis of the jaw (ONJ)
  - Pericoronitis or third-molar pain
  - Surgical postoperative osteitis
(iii) Urgencies and pathologies unresponsive to medical therapy (treatment after a diagnostic test)
  - Abscess or localized bacterial infection
  - Biopsy of abnormal tissue
  - Dental trauma with avulsion/luxation
  - ONJ unresponsive to medical therapy
  - Pericoronitis or third-molar pain (resistant to medical therapy)
  - Surgical postoperative osteitis (resistant to medical therapy)
  - Removal of nonabsorbable sutures
(iv) Emergencies (need for immediate treatment)
  - Diffused soft tissue infection with intraoral or extraoral swelling that potentially compromise patient's airway
  - Uncontrolled bleeding

If patients had no recent X-ray examinations, they were performed in the clinic. If patients described symptoms compatible with pulpitis, they were referred to the Endodontics unit, present in the same building as the Oral Surgery unit. Conversely, if the patient's symptoms were compatible with surgical pathologies of the oral cavity, they were referred to the Oral Surgery unit.

### 2.2. Staff Management

Four units were involved in each patient management:

1. One administrative staff member
2. One nursing staff member outside the operative area
3. One dental assistant inside the operative area
4. One oral surgeon

All operators followed the same prevention rules: washing hands with alcohol-based hand sanitizer for at least 20 seconds, before and after each treatment, and limiting contact with surfaces and devices as much as possible; avoiding to touch their faces, including the eyes, nose, and mouth; and avoiding the use of any personal accessory (bracelets, rings, watches, etc.). Sterile preparation criteria should be applied on every step of the clinical practice, including the operator dressing-undressing routine. Staff members were dressed in personal protective equipment (PPE), as described in Table 1. Staff members underwent the swab test for SARS-CoV-2 every two weeks.

### 2.3. Patient Management

**Figure 1:**
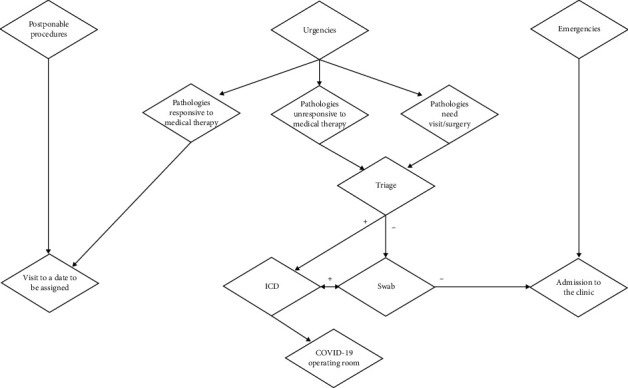
Summary flow chart of patient management for admission to clinic.

Patients received remote therapeutic indications to resolve symptoms whenever possible. When medical therapy was not indicated or had proven to be ineffective, patients had to visit the hospital. It was fundamental to contact the patient by phone and ask a few questions about their state of health, especially if they had a fever (>37.5°C), cold, cough, breathing difficulties, muscle pain, and headache, which had arisen in the last 14 days. In addition, it was necessary to ask the patient if they had visited areas at risk, if they had been in contact with infected people or people coming from an infectious outbreak, or with people who had symptoms, in the last 14 days. If the patient had one of the above symptoms or contacts, they were referred to the infection control department (ICD). If the patient's condition required intervention at the clinic, they underwent a swab test for SARS-CoV-2 prior to access to the clinic (24 hours needed for results). For emergencies, patients were treated without testing. Any positive patients were referred to ICD and then treated in the operating room dedicated to hospitalized COVID-19 patients. Patients negative to the SARS-CoV-2 test were admitted to the clinic, and body temperature was measured on arrival with an electronic thermometer without direct contact. A questionnaire was submitted, with the same questions asked by telephone, in order to confirm remote triage. Summary flow chart is available in Figure 1.

### 2.4. Prevention of Cross Infection in Nonclinical Areas

Staff, patients, and accompanying persons must respect the interpersonal distance of one meter in these areas. Patients numbers and accompanying people should be dramatically reduced; for example, patients had to wait outside the hospital. Children and nonautonomous patients were accompanied only by one person. Promiscuous material from the waiting room was removed, and chairs were covered with disposable towels. A disinfectant dispenser was installed to allow patients to sanitize their hands. Personal items were not allowed in clinical areas. It was necessary to disinfect handles, devices, and surfaces after each contact and to ventilate the rooms frequently.

### 2.5. Prevention of Cross Infection in Clinical Area

Only necessary tools were placed on the shelves. It was fundamental to protect the dental chair and all the tools with disposable films. Dentist and assistant sanitized their hands before and after the use of PPE, as described in Table 1. It was recommended to use mouth rinse containing oxidative agents such as 1% hydrogen peroxide or 0.2% povidone, instead of chlorhexidine, considering the susceptibility of coronaviruses [10, 12, 13]. In case of tooth extraction, minimally invasive procedures were preferred. The use of high-speed drilling was limited, but, when necessary, rotating instruments were used on contra-angle with low-speed drilling and minimum water irrigation. Piezoelectric devices and cautery have not been used. To limit patient access to the clinic, multiple therapies have been performed, and fast absorbable suture and hemostatic materials were mandatory. At the end of the treatment session, the disposable films were removed with clean gloves, all instruments and surfaces were disinfected with suitable disinfectants (e.g., isopropyl alcohol and hydrogen peroxide), and the disposable material was properly disposed. The rooms were ventilated as much as possible. The operators washed their hands for at least 1 minute and then applied 60% hydroalcoholic solution.

## 3. Results

From February 24 to May 3, 2020, 296 patients contacted the Oral Surgery Unit by phone. 166 patients were managed remotely: among these, 46 patients required postponable procedure and 120 patients received indications for medical therapy (painkillers, antibiotics, and mouthwashes) which proved to be effective (Table 2). Of the 130 patients admitted, 4 had responded positively to at least one triage question and were referred to ICD. The Institute of Microbiology of our University sent the results 15 hours (mean value, range 10 to 19 hours) after the swab was run. All swabs were negative. The mean age of admitted patients was 40.3 years (range 16–74); 76 were males and 54 females with a male to female ratio of 1.4 : 1. 82 patients (63%) came from the Province of Catanzaro, and the others from other provinces of the Calabria Region. One patient was admitted two times (biopsy and bleeding); therefore, it has been counted twice. List of outpatient oral surgery procedures performed is available in Table 3. The main reason for admission to the clinic was removal of nonabsorbable sutures (oral surgery procedures performed before the emergency phase), followed by abscesses, and biopsy of abnormal tissue. In addition, 34 radiographic examinations were performed in the clinic. On authors' knowledge, no cases of positivity to SARS-CoV-2 have been detected among patients and staff members.

## 4. Discussion

SARS-CoV-2 can be transmitted from person to person mainly by droplets or saliva [14, 15]. Indeed, all procedures that may airborne the virus through aerosol should be indicated as potential transmission routes. The main source of transmission is certainly the symptomatic COVID-19 patients, but asymptomatic patients and patients in their window period are also carriers [16–18]. The incubation period of COVID-19 has been reported to be 1 to 14 days (5 to 6 days on average) [19]. The surgical pathology of the oral cavity is highly specific, and the oral surgeon can be exposed to microorganisms present in the mouth and respiratory tract during clinical examinations and procedures.

In March 2020, the Italian Ministry of Health ordered the suspension of all nonurgent outpatient activities (including dentistry and oral and maxillofacial surgery) in hospitals and clinics of the public health system, until further notification according to the situation of pandemic. All Italian professional dental and medical associations equally recommended the suspension of nonurgent activities in private clinics [11].

Our University Hospital (“Mater Domini” Hospital, Catanzaro, Italy) was chosen as the COVID-19 Reference Hospital for the Calabria Region because it is the only hospital with an intensive care unit equipped for extracorporeal membrane oxygenation (ECMO).

In this difficult time, the availability of PPE in our hospital has allowed us to replace private oral healthcare. To face up this new scenario, our department needed to develop consistent guidelines to ensure appropriate patient care.

This study describes the experience of an oral surgery unit during the COVID-19 pandemic. The staff handled 298 calls: 46 patients asked for information on previously scheduled appointments, 120 patients called because they had symptoms and were managed remotely with medical therapy, and 130 patients needed clinical intervention to resolve the symptomatology. After remote triage, we prescribed 125 swabs. The results were available after an average of 15 hours (range 10 to 19 hours). Four patients responded positively to at least one of telephone triage questions and were referred to ICD for investigation. All swabs tested negative, but because of sampling error or biology of the disease, we could not rule out the presence of false negatives. Only one patient returned to the clinic after a biopsy for uncontrolled bleeding, and as an emergency he was not subjected to a swab before admission. Sixty-four procedures were classified as nondeferrable urgencies. For all 65 accesses to the clinic, remote triage was repeated in written form. More than half of these patients came from the province where the clinic is located.

The staff correctly used PPE and disposable material even during sanitization between one patient and another as described above. The purpose of using the double mask was to preserve the use of filtered masks, given the limited availability. In this way, only the surgical mask was disposed after each patient and the filtered mask was used for the entire shift by the operator.

Remote consultation was improved to check for the urgency of a patient's condition and prescribe a treatment plan. This management has been very effective considering that most of ONJ and third-molar pain have been managed through the prescription of medical therapy. Likewise, remote consultations made it possible to reduce the number of patients and the number of times they had access to the clinic [20]. Furthermore, the application of a scrupulous triage protocol and the correct use of PPE allowed no contagion among patients and staff members.

However, both the pandemic and the application of these measures have reduced the number of procedures performed in the clinic. For this reason, the need for oral healthcare might grow in the following weeks. Possible health consequences resulting from fear of hospitals and clinics should not be overlooked. Understanding the present situation may be helpful in terms of predicting future health needs [21].

Given the experience gained in the management of outpatient oral surgery during the COVID-19 pandemic, the authors recommend the following:

- Performing a remote triage and repeating it when the patient arrives at the clinic
- Prescribe the SARS-CoV-2 diagnostic test before admission to the clinic if the patient's condition allows surgeons to wait 24 hours
- Use PPE correctly and scrupulously
- Perform the presurgical preparation as quickly as possible
- Use of a room with a surgical table/dental chair for a single patient
- Apply surgical techniques that minimize operating times and aerosol production
- Use resorbable sutures and hemostatic material
- Take advantage of telemedicine for postoperative management whenever possible

The goal was to identify the potentially infected patients before leaving home and refer them to the ICD for suspected COVID-19, but if urgent care was needed, they underwent a swab test for SARS-CoV-2 prior to access to the clinic. The protection of healthcare workers, the overall reduction in the time spent by patients in the clinic, and the search for asymptomatic SARS-CoV-2 positive patients remain the fundamental measures to reduce the risk of cross infection.

Restrictions imposed by governments made it possible to stop the advance of SARS-CoV-2 outbreak, but there is always the possibility that the virus can recur with new outbreaks. In order to restart elective activities in a sustainable way, it may be useful to test patients before entering the clinic [22]. Without epidemiological studies ascertaining the presence of immunity in the population, we must consider that most of the population is still susceptible to infection. Therefore, it will probably be necessary to continue carrying out outpatient activities in the same way as described in this article.

## 5. Conclusion

The lesson learned from the COVID-19 pandemic was vital in developing protocols and guidelines to limit contagion, both for healthcare professionals and patients. Within the limitation of this study, our experience showed the impact of the COVID-19 pandemic on outpatient oral surgery activities. The protocols developed will allow us to act proactively in the next phase of eased restrictions, but the medical community must stay alert.

## Figures and Tables

**Table 1:** Personal protective equipment: various scenarios after admission to the clinic.

	Nonclinical areas	Clinical area—nonaerosol generating procedures	Clinical area—aerosol generating procedures
Cap	✓	✓	✓
Face shield			✓
Filtered mask^§^		✓	✓
Gloves	✓	✓	✓
Protective glasses		✓	✓
Protective waterproof clothing			✓
Shoe cover		✓	✓
Surgical mask	✓	*∗*	*∗*

^*∗*^In order to reuse filtered masks, they were covered with a surgical mask. The filtered masks were disposed of at the end of the work shift. ^§^N-95 masks, National Institute for Occupational Safety and Health, USA, or FFP2/FFP3-standard masks, European Union.

**Table 2:** Patients managed remotely.

	Number of patients
*Postponable procedures*	46
Periodic oral examinations and recall visits	23
Extraction of asymptomatic teeth	16
Implantology	4

*Pathologies responsive to medical therapy*	**120**
ONJ	22
Pericoronitis or third-molar pain	79
Surgical postoperative osteitis	19
*Total*	**166**

**Table 3:** List of outpatient oral surgery procedures performed.

Category of procedures	Number of patients	Treatment
*Emergencies*	**1** ^**∗**^	
Diffused soft tissue infection with intraoral or extraoral swelling that potentially compromise patient's airway	—	—
Uncontrolled bleeding	1^*∗*^	Application of hemostatic material and suture (1)

*Urgencies*	**129**	
Abscess, or localized bacterial infection	12	Abscess drainage, tooth extraction, and medical therapy (9) Abscess drainage, medical therapy, and referred to endodontic unit (3)
Biopsy of abnormal tissue	26	Incisional biopsy (18) Excisional biopsy (6) Follow-up (2)
Dental trauma with avulsion/luxation	5	Retainer application (4) Tooth extraction (1)
ONJ unresponsive to medical therapy	7	Surgical debridement and medical therapy (7)
Pericoronitis or third-molar pain (resistant to medical therapy)	19	Tooth extraction (15) Ulotomy (4)
Surgical postoperative osteitis (resistant to medical therapy)	8	Curettage and medical therapy (8)
Removal of nonabsorbable sutures	52	Suture removal and wound irrigation (52)

^*∗*^The patient underwent biopsy during the pandemic.

## Data Availability

Additional data are available from the corresponding author upon reasonable request.

## References

[1] Xu Z., Shi L., Wang Y. (2020). Pathological findings of COVID-19 associated with acute respiratory distress syndrome. *The Lancet Respiratory Medicine*.

[2] Yang X., Yu Y., Xu J. (2020). Clinical course and outcomes of critically ill patients with SARS-CoV-2 pneumonia in Wuhan, China: a single-centered, retrospective, observational study. *The Lancet Respiratory Medicine*.

[3] Bennardo F., Buffone C., Giudice A. (2020). New therapeutic opportunities for COVID-19 patients with Tocilizumab: possible correlation of interleukin-6 receptor inhibitors with osteonecrosis of the jaws. *Oral Oncology*.

[4] Spagnuolo G., De Vito D., Rengo S., Tatullo M. (2020). COVID-19 outbreak: an overview on dentistry. *International Journal of Environmental Research and Public Health*.

[5] World Health Organization (2020). Coronavirus disease 2019 (COVID-19). https://www.who.int/emergencies/diseases/novel-coronavirus-2019/situation-reports/.

[6] Peng X., Xu X., Li Y., Cheng L., Zhou X., Ren B. (2020). Transmission routes of 2019-nCoV and controls in dental practice. *International Journal of Oral Science*.

[7] Meng L., Hua F., Bian Z. (2020). Coronavirus disease 2019 (COVID-19): emerging and future challenges for dental and oral medicine. *Journal of Dental Research*.

[8] From The New York Times (2020). The workers who face the greatest coronavirus risk. https://www.nytimes.com/interactive/2020/03/15/business/economy/coronavirus-worker-risk.html.

[9] van Doremalen N., Bushmaker T., Morris D. H. (2020). Aerosol and surface stability of SARS-CoV-2 as compared with SARS-CoV-1. *New England Journal of Medicine*.

[10] Fiorillo L., Cervino G., Matarese M. (2020). COVID-19 surface persistence: a recent data summary and its importance for medical and dental settings. *International Journal of Environmental Research and Public Health*.

[11] Bennardo F., Buffone C., Fortunato L., Giudice A. (2020). COVID-19 is a challenge for dental education—a commentary. *European Journal of Dental Education*.

[12] Kampf G., Todt D., Pfaender S., Steinmann E. (2020). Persistence of coronaviruses on inanimate surfaces and their inactivation with biocidal agents. *Journal of Hospital Infection*.

[13] Fiorillo L. (2019). Chlorhexidine gel use in the oral district: a systematic Review. *Gels*.

[14] Rothan H. A., Byrareddy S. N. (2020). The epidemiology and pathogenesis of coronavirus disease (COVID-19) outbreak. *Journal of Autoimmunity*.

[15] Jiang F., Deng L., Zhang L., Cai Y., Cheung C. W., Xia Z. (2020). Review of the clinical characteristics of coronavirus disease 2019 (COVID-19). *Journal of General Internal Medicine*.

[16] Chan J. F.-W., Yuan S., Kok K.-H. (2020). A familial cluster of pneumonia associated with the 2019 novel coronavirus indicating person-to-person transmission: a study of a family cluster. *The Lancet*.

[17] Rothe C., Schunk M., Sothmann P. (2020). Transmission of 2019-nCoV infection from an asymptomatic contact in Germany. *New England Journal of Medicine*.

[18] Herron J. B. T., Hay-David A. G. C., Gilliam A. D., Brennan P. A. (2020). Personal protective equipment and Covid 19- a risk to healthcare staff?. *British Journal of Oral and Maxillofacial Surgery*.

[19] Backer J. A., Klinkenberg D., Wallinga J. (2020). Incubation period of 2019 novel coronavirus (2019-nCoV) infections among travellers from Wuhan, China, 20–28 January 2020. *Eurosurveillance*.

[20] Giudice A., Bennardo F., Antonelli A., Barone S., Fortunato L. (2020). COVID-19 is a new challenge for dental practitioners: advice on patients’ management from prevention of cross infections to telemedicine. *The Open Dentistry Journal*.

[21] Yakubov D., Ward M., Ward B., Raymond G. F., Paskhover B. (2020). Opinion: an increase in severe, late dental complications might result from reliance on home dental remedies during the COVID-19 pandemic. *Journal of Oral and Maxillofacial Surgery*.

[22] Giudice A., Antonelli A., Bennardo F. (2020). To test or not to test? An opportunity to restart dentistry sustainably in “COVID‐19 era”. *International Endodontic Journal*.

